# Mobilizing the Power of Lived/Living Experiences to Improve Health Outcomes for all

**DOI:** 10.1111/hex.70212

**Published:** 2025-03-18

**Authors:** Ambreen Sayani, Linda Monteith, Anam Shahil‐Feroz, Diya Srinivasan, Isra Amsdr, Fatah Awil, Emily Cordeaux, Victoria Garcia, Ryan Hinds, Tara Jeji, Omar Khan, Bee Lee, Mursal Musawi, Jill Robinson, Staceyan Sterling, Dean Wardak, Kelly Wu, Mohadessa Khawari, Meghan Gilfoyle, Alies Maybee

**Affiliations:** ^1^ Women's College Research Institute, Women's College Hospital Toronto Ontario Canada; ^2^ Equity Mobilizing Partnerships in Community (EMPaCT), Women's College Hospital Toronto Ontario Canada; ^3^ Dalla Lana School of Public Health University of Toronto Ontario Canada; ^4^ Arthur Labatt Family School of Nursing University of Western Ontario London Ontario Canada; ^5^ Patient Advisors Network (PAN) Toronto Ontario Canada

**Keywords:** CEn‐HEA, community engaged health equity analysis, diversity, EMPaCT, equity, health equity, health equity analysis, inclusion, patient engagement, patient‐oriented research, power

## Abstract

**Introduction:**

Health Equity Assessments (HEAs) are decision‐support frameworks or tools used to evaluate the equity impacts of policies, programmes and initiatives. However, HEAs are often conducted without meaningful engagement from the individuals and communities most affected by health inequities. This lack of social participation limits the relevance and effectiveness of HEAs, leaving systemic inequities unaddressed and opportunities for impactful change unrealized. An alternative is to involve people with diverse lived/living experiences in conducting and offering HEAs—so that people most impacted, and most excluded by decision‐making can offer recommendations to improve the way they access and utilise care.

**Methods:**

Equity Mobilizing Partnerships in Community (EMPaCT) is a scalable, participatory citizen engagement model that integrates lived/living experiences into the HEA process. EMPaCT's Five Steps to a Community‐Engaged Health Equity Assessment (CEn‐HEA) was co‐designed with community members typically excluded from decision‐making. This process fosters psychological safety, trust‐building, and power‐sharing between underserved communities and decision‐makers. The CEn‐HEA systematically analyzes inequities across downstream (individual), midstream (community), and upstream (structural) levels to generate actionable, equity‐focused recommendations.

**Results:**

The EMPaCT CEn‐HEA framework produces context‐specific recommendations that address immediate project needs while advancing long‐term, systemic change. The framework is a participatory process that centres community voices, builds trust, amplifies lived/living expertise, and fosters equity‐driven decision‐making that can lead to measurable improvements in healthcare policies, programmes, and practices.

**Conclusion:**

In this paper, we examine the challenges and opportunities associated HEAs; introduce EMPaCT's CEn‐HEA framework as a co‐designed, innovative, and community‐engaged approach to health equity analysis; and discuss methods for measuring and evaluating the health equity impacts of these efforts.

**Patient or Public Contribution:**

Patient and community involvement were central to the design, development and implementation of this project and resulting manuscript. Equity Mobilizing Partnerships in Community (EMPaCT), including its Community‐Engaged Health Equity Assessment (CEn‐HEA) framework, was co‐created with diverse patient partners who have lived/living experiences of health inequities. In the preparation of this manuscript, patient partners were involved in codesign sessions to define the focus, structure and language of the manuscript. They collaborated in discussions to refine key concepts, articulate challenges and highlight solutions that are grounded in their lived realities. In the preparation of this manuscript, patient partners reviewed early drafts, contributed feedback to ensure accessibility and relevance of the content and shaped the actionable recommendations. This manuscript reflects EMPaCT's commitment to justice, inclusion and meaningful change.

## 1 Introduction

Health Equity Assessments (HEA) are tools and decision‐support frameworks used to systematically understand and evaluate the potential health equity impacts of policies, programmes, or projects. HEAs can expose underlying health inequities and identify mitigation strategies to improve health equity within the scope of an initiative across population groups, with an emphasis on those who are structurally underserved and experiencing social disadvantage [1, 2, 3]. Other terms used synonymously with HEA include Health Equity Audit, Health Impact Assessment, Health Equity Impact Assessment and Equity‐Focused Health Impact Assessment [4, 5].

The World Health Organization [5, 6] emphasizes the utility of HEAs as an essential tool for evaluating and addressing health inequities, promoting equitable access to health resources, and advancing policies, programmes and interventions that create fair and inclusive health outcomes for all population groups. HEAs are used widely by international organisations, governments, and health and social care organisations. For instance, HEAs have been used in Australia for regional land‐use planning and public health interventions [7] in New Zealand to assess the impacts of transport policies on health equity [8]; in the United Kingdom to guide spatial planning efforts [9] and to incorporate health equity considerations into environmental and public health policies [3]. In Canada, HEA tools have been used to enhance mental healthcare services and policy planning [1].

Despite the widespread use of HEAs, several limitations hamper their ability to make significant progress towards reducing avoidable differences in health and promoting fairness and justice in health outcomes—key tenets of advancing health equity [3, 10, 11]. Some of these limitations include inconsistent conceptual frameworks, inadequate consideration of broader policy environments, poor data quality, challenges in stakeholder engagement, and a focus on downstream factors rather than the fundamental causes of health inequities [3, 4, 12]. Further, global perspectives,‐ particularly from Indigenous communities, which emphasize relational approaches to power, shared resource stewardship, collective decision‐making, interdependence, and long‐term sustainability [13], can directly contradict the individualistic and Western‐centric paradigms often underlying HEA frameworks.

In this paper, we (i) describe the challenges and opportunities with HEAs as they are frequently used; (ii) introduce a novel approach to HEAs that integrates the lived/living experiences of diverse community members called Equity Mobilizing Partnerships in Community (EMPaCT's) Five Steps to a Community‐Engaged Health Equity Assessment (CEn‐HEA); and (iii) reflect on how the health equity impact of such efforts can be measured and evaluated.

## 2 Challenges and Opportunities With Health Equity Assessments (HEAs)

### 2.1 Conceptual Tensions

HEAs are designed to explore and address the potential impacts of policies, programmes, and projects on health inequities. At a baseline, HEAs aim to avoid unintentionally worsening existing health inequities. When effectively implemented, they offer an opportunity to foster fairness and advance social justice. However, the lack of clear and standardized definitions of equity, inequity, and inequality in HEA guidelines and reports has led to inconsistent adoption and variable implementation [3].

For instance, HEAs often rely on explanatory models that extrapolate from social phenomena to emphasize individual‐level actions rather than social causes, a tendency known as **methodological individualism** [14, 15]. Using obesity as an example, health inequities are frequently framed as the result of personal choices, such as unhealthy eating or physical inactivity, rather than the systemic conditions that constrain those choices, such as food insecurity, unaffordable healthy food options, unsafe neighbourhoods, or targeted marketing of unhealthy products [16]. This framing shifts attention away from structural determinants onto the individual, leading to fragmented equity‐promoting efforts.

This focus on individual behaviours often gives rise to **behavioural determinism**, where inequities are identified as consequences of personal risk factors that need intervention [14, 15]. In the context of obesity, interventions frequently emphasize educating individuals to “make better choices” or adopt healthier lifestyles while neglecting the structural barriers that shape these choices.

Compounding this is the phenomenon of **lifestyle drift**, where interventions initially designed to address systemic inequities gradually shift toward promoting individual behaviour change instead [17, 18]. For example, instead of targeting upstream factors such as urban planning decisions that create food deserts or economic policies that drive poverty, interventions often default to encouraging healthier eating and exercise. This drift reinforces a narrow focus on individual responsibility while leaving systemic inequities unchallenged.

**Figure 1:**
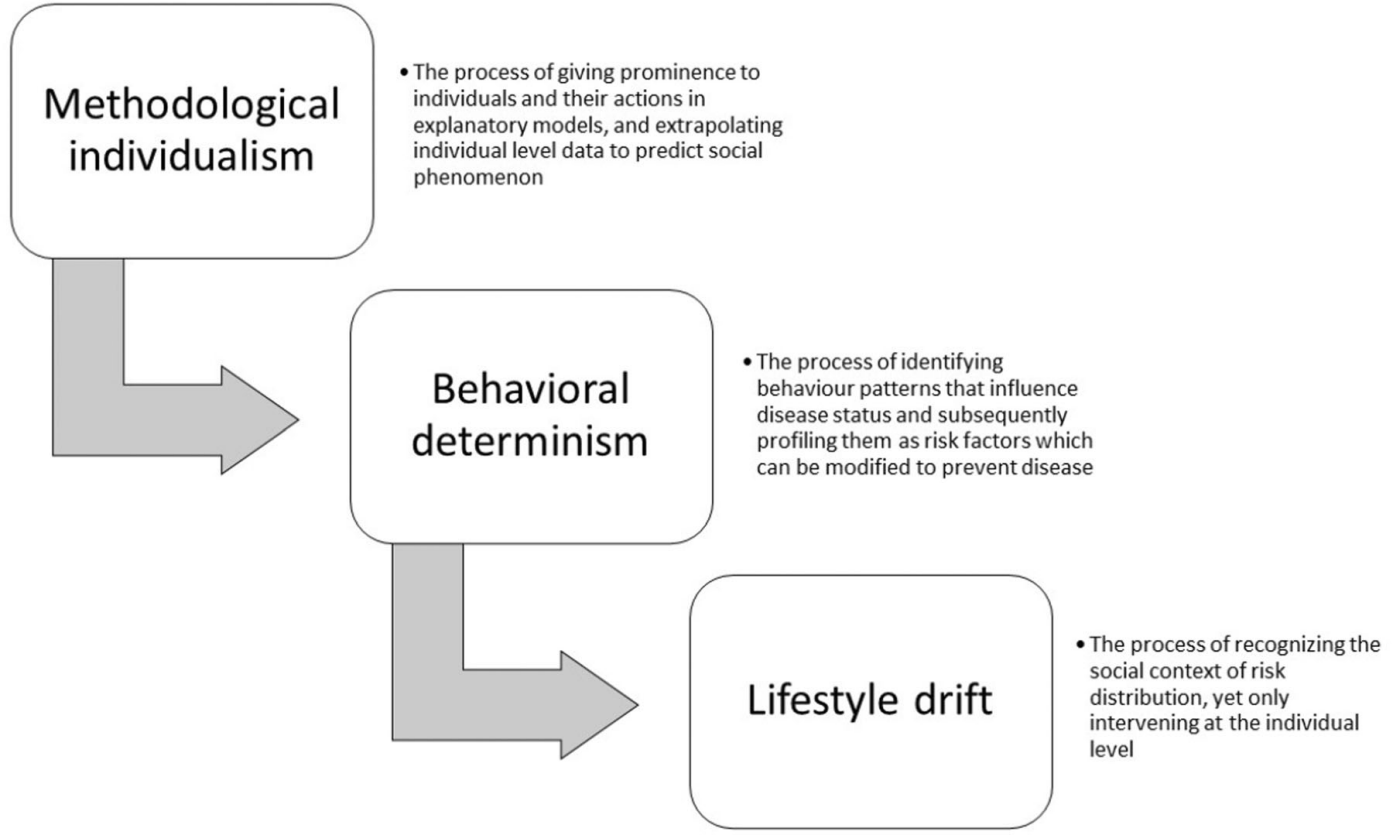
Causal pathways in health systems design thinking that perpetuate health inequities.

When explanatory models emphasize **methodological individualism**, prioritize **behavioural determinism**, and succumb to **lifestyle drift**, they fail to address the fundamental or root causes of inequities [12] (see Figure 1). Consequently, health system policies and programmes fall short in reducing gaps in care and perpetuate inequities rather than mitigating them [19]. To fulfil their potential, HEAs must centre systemic and structural drivers of inequities, ensuring that assessments and interventions address these root causes and promote sustainable, transformative change.

### 2.2 Data Availability and Interpretation

HEAs rely heavily on the availability of robust, high‐quality disaggregated population health data to identify and address health inequities. However, significant gaps remain in the accessibility and completeness of data on the social determinants of health, particularly for structurally underserved populations [2, 4]. These gaps can result from systemic barriers in data collection, reporting, and sharing practices, which often exclude marginalized groups. Specifically, data related to risk exposures, living conditions, income inequality, housing security, and education—are frequently limited or missing [20]. Such gaps in data hinder the adoption of a **life course perspective** using a HEA, which is critical for addressing health inequities comprehensively [21]. A life course perspective examines how social, economic, and environmental exposures accumulate and interact over an individual's lifetime to influence health outcomes. By focusing on health trajectories from childhood through adulthood and into older age, this approach highlights how inequities are shaped by intersecting factors such as housing policies, educational access, and employment opportunities over time. Incorporating this perspective enables HEAs to move beyond addressing immediate or downstream factors and instead target the structural conditions that underpin inequities. To address these data limitations requires improved data collection methods and a concerted effort to operationalise and synthesize evidence on the root causes of health inequities, including macroeconomic policies, historical injustices, systemic racism, and the effects of globalization and neoliberalism [3, 22]. Neoliberalism refers to reducing the role of government by promoting individual responsibility and putting profits over people's wellbeing [22].

### 2.3 Beneficiary Engagement

The term ‘stakeholder’ engagement traditionally means groups with vested interests in a project. It is often criticized for reflecting power hierarchies and colonial histories, privileging influence over equity [23, 24, 25]. While the term ‘interest‐holder’ is a more precise acknowledgement of diverse perspectives [26], we believe it lacks the explicit relational and outcome‐oriented focus necessary for transformative engagement. We prefer ‘beneficiary’ engagement with its explicit link to ‘benefit’, emphasizing the principles of upfront investments in relationships and collaborative spaces. Beneficiary engagement ensures time is dedicated to fostering trust, creating opportunities for shared listening and learning, and reaching a collective point of problem‐posing [27] before solution‐building begins. Through this process individuals with lived/living experience, healthcare providers, and system administrators are co‐creators, ensuring that all parties are enriched through equitable and meaningful collaboration [28, 29].

By prioritizing these principles, beneficiary engagement directly supports the goals of the Quintuple Aim (a framework designed to transform healthcare systems by improving population health, enhancing patient and provider experiences, reducing healthcare costs, advancing health equity [30]). It holds the potential to address the determinants of health through sustained, interdisciplinary, inclusive partnerships and collaboration [3].

### 2.4 HEA Impact Evaluation

Evaluating the impact of HEAs is complex due to the traditional focus on immediate, downstream factors rather than upstream determinants (e.g., sociohistorical conditions and economic policies). This narrow application of HEA in practice, accompanied by a lack of standardized impact metrics, hinders the systematic evaluation of impact effectiveness [3]. HEAs need to incorporate a comprehensive impact evaluation framework to ensure meaningful outcomes. For example, immediate outcomes of beneficiary‐engaged HEAs can include metrics of community capacity‐building, tracking of interdisciplinary partnerships and degree of influence in health system decision‐making [29]. Intermediate outcomes should focus on tracking equity‐promoting changes to programmes, policies and metrics that demonstrate improved access to healthcare for structurally underserved groups [2]. The long‐term impacts of HEAs must sustainably enhance the health of the most underserved groups, narrow health gaps between population groups, and aim to reduce health gradients [19].

These tensions and limitations underscore the need for a new approach to HEAs—one that emphasizes equity‐promoting public engagement, integrates diverse perspectives and offers practical tools to track and measure impact. The following section introduces a framework co‐designed to address these gaps by fostering meaningful collaboration, centering lived/living experience and co‐creating accountability mechanisms that promote sustainable action towards health equity.

## 3 Community‐Engaged HEAs (CEn‐HEAs)—the EMPaCT Model

EMPaCT is a model of public and citizen engagement that centres the perspectives of diverse community members in health and social care decision‐making to advance health equity [31]. The model was co‐designed through iterative collaboration with community members, healthcare providers and decision‐makers, drawing on lived/living experiences and equity‐driven principles [28] to create a participatory model for health equity analysis. Developed in Canada, the model reflects the contextual realities of urban settings and healthcare systems, focusing on addressing structural inequities [31]. Designed with scalability in mind, EMPaCT incorporates a phased approach for broad implementation based on core principles, adaptable to varied contexts and can be applied effectively in diverse geographic, policy and organizational settings [31]. This model addresses the shortcomings of traditional engagement practices [28], which often exclude marginalized perspectives, by fostering safe inclusive spaces for meaningful engagement. Grounded in both the published literature [32] and our collective experience as a group we have co‐developed a list of key principles of meaningful engagement (Table 1) that guide this work. Through intentional power‐sharing, EMPaCT focuses on beneficiary engagement, emphasizing the collective role of all partners in shaping decisions that impact their everyday lives. Details of EMPaCT's co‐creation [31], membership [29, 31] and power sharing mechanisms can be read elsewhere [33].

**Table 1:** Meaningful engagement—conceptual definition and key principles.

**Meaningful engagement is defined as** a collaborative process that fosters trust, inclusivity and shared accountability by centering the voices, experiences and expertise of all partners, including individuals with lived/living experiences. Rooted in beneficiary engagement, it prioritizes equitable relationships, mutual learning and the co‐creation of decisions that address collective priorities and produce actionable outcomes.
**Ten key principles of meaningful engagement are:** 1. **Include Diverse Perspectives:** Actively involve voices from structurally marginalized or underrepresented groups and value all contributions equally from the outset. 2. **Create Culturally Safe and Comfortable Spaces:** Establish environments where participants feel respected, secure, and valued, enabling authentic participation and collaboration. 3. **Build Trust:** Cultivate genuine relationships through respect, consistency, and follow‐through on commitments to establish a foundation for collaboration. 4. **Communicate Transparently:** Clearly define goals, roles, expectations, and decision‐making processes to promote mutual understanding and clarity. 5. **Foster Reciprocity:** Nurture relationships that promote mutual learning, knowledge‐sharing, and reciprocal value for all partners involved. 6. **Value Lived/Living Experiences:** Recognize lived/living experiences as integral evidence that complements technical or academic expertise. 7. **Strengthen Capacity:** Enhance participants' skills, knowledge, and resources to support their sustained involvement and leadership in engagement processes. 8. **Share Power Equitably:** Distribute decision‐making authority fairly, promoting balanced influence and shared responsibility for outcomes. 9. **Promote Accountability:** Implement mechanisms to track progress, reflect on contributions, and translate insights into meaningful actions. 10. **Align Priorities for Impact:** Tailor engagement strategies to reflect participants' values and needs, creating actionable and context‐specific outcomes.

At the core of EMPaCT is a co‐designed, five‐step **Community‐Engaged Health Equity Analysis (CEn‐HEA).** This framework systematically identifies, examines and addresses health inequities at downstream (individual health), midstream (community‐level factors) and upstream (structural determinants) levels [21]. Each step of the CEn‐HEA framework reflects key principles of meaningful engagement—centering lived/living experience, creating safe spaces and embedding accountability into every stage.

**Figure 2:**
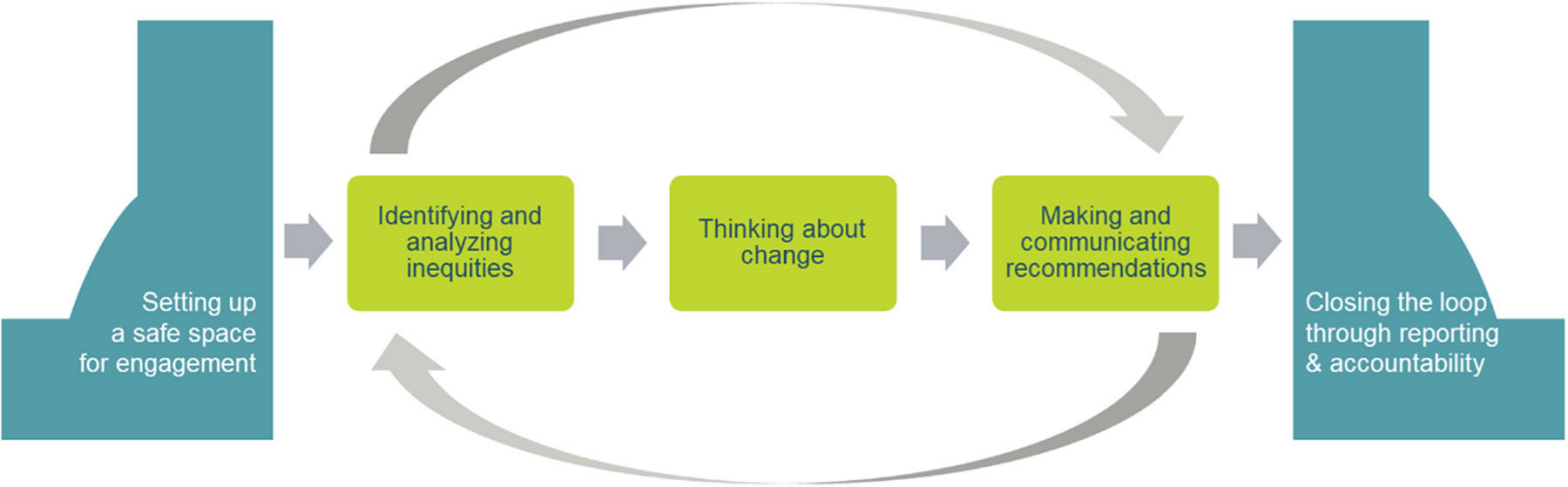
EMPaCT's five steps to a community‐engaged health equity analysis CEn‐HEA.

**Figure 3:**
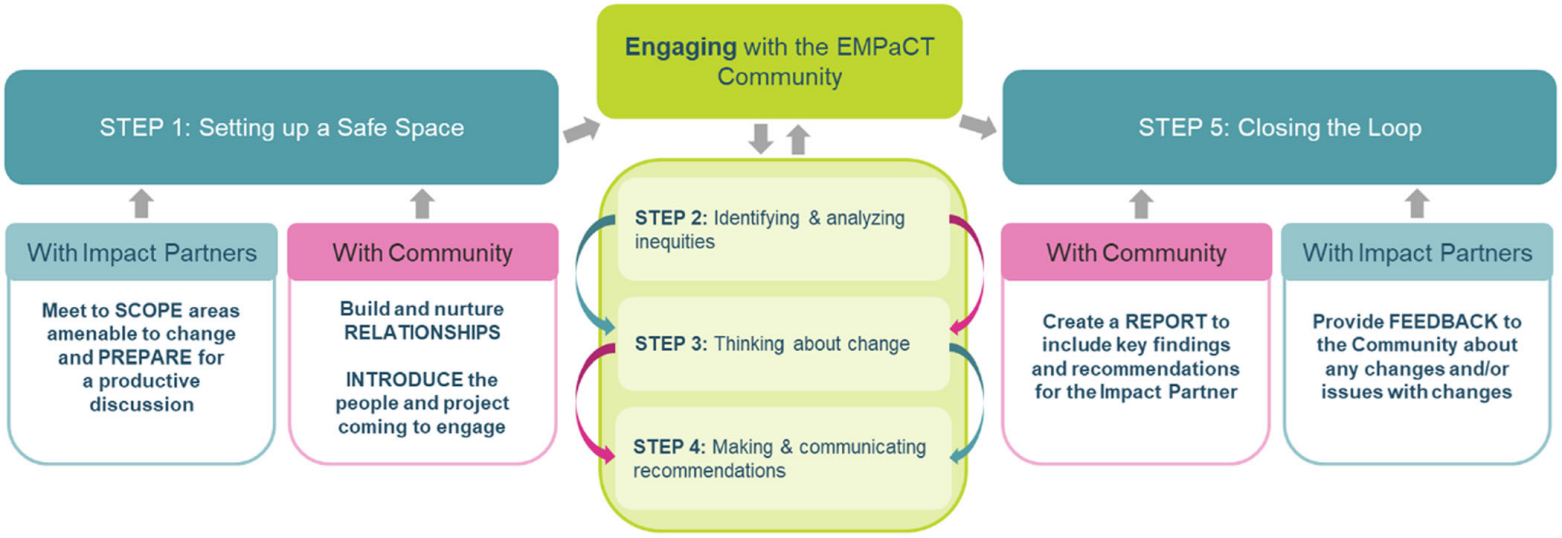
EMPaCTs community‐engaged health equity analysis CEn‐HEA process map.

These steps are illustrated in Figure 2. The processes constituting each step are described in detail below and in Figure 3. to support those seeking to implement a similar approach within their context.

### 3.1 Step 1: Setting up Safe Spaces for Inclusive Engagement

Psychological safety—a sense of trust, mutual respect and assurance that participants can engage openly without fear of judgement or harm—is the cornerstone of meaningful engagement (see Table 1). This step serves as both the foundation and the front bookend of the CEn‐HEA, enabling subsequent steps to be grounded in trust and inclusivity. Safe spaces are essential for fostering authentic collaboration and co‐learning between community members and decision‐makers, referred to as ‘Impact Partners’ due to their critical role in implementing change Sayani et al. [25].

We have learned that psychological safety in engagement offers two distinct yet complementary experiences for engagement participants:

1. **For community members**, safety provides the freedom to share lived/living experiences, voice concerns and engage fully without fear of tokenism, dismissal or retribution.
2. **For Impact Partners**, safety creates a space to reflect on systemic blind spots, confront challenges and act on community feedback constructively and without defensiveness.

Creating psychological safety requires intentional preparation and processes:

- **Coaching and preparation for Impact Partners**: This includes two to three preparatory meetings designed to align project goals with community priorities, identify areas amenable to change and equip participants with skills for active listening, collaborative dialogue and respectful, effective engagement. A maximum of three Impact Partners are allowed to participate, of whom at least one must be a key project decision‐maker. These processes shift traditional power dynamics. EMPaCT defines the terms of engagement, including meeting formats, timing and compensation while health system decision‐makers seek EMPaCT's expertise.
- **Trust‐building among community members**: Priority is given to dedicated time for connection, collaboration and trust‐building among community members. New members are onboarded in pairs or smaller groups to create a sense of ease and belonging. Trust cultivated through existing relationships, or ‘derived rapport’, [34] strengthens interpersonal bonds so that participants feel better supported and valued.

These strategies and behaviours become group norms critical for sustaining psychological safety.

This step prepares all partners for equitable collaboration and sets the tone for sustained engagement and accountability throughout the entire process.

### 3.2 Steps 2–4: Engaging With the EMPaCT Community

At the centre of EMPaCT's CEn‐HEA process are Steps 2 to 4, forming a structured 2‐hour virtual engagement. Community members and Impact Partners collaboratively assess inequities, identify actionable changes, and develop equity‐driven recommendations in a trust‐based environment. These steps form an iterative cycle of co‐learning, and collaborative problem‐solving, transitioning from analysis to action to promote meaningful and sustained equity‐promoting outcomes.

### 3.3 Step 2: Identifying and Analyzing Inequities

Impact Partners and community members explore how the proposed project interacts with inequities across multiple levels. Downstream inequities include immediate impacts on individuals, such as access to health services, while midstream inequities focus on community‐level factors like neighbourhood infrastructure and social networks. Upstream inequities examine structural drivers such as systemic racism, institutional policies and historical injustices. By centering lived/living experience as critical evidence, this step surfaces inequities that may otherwise remain invisible through population‐level data. It also highlights who benefits from the project, who may be excluded, and the potential unintended consequences of inaction or misaligned interventions from the perspective of those who are most impacted. For all participants these discussions (manuscript forthcoming) create a space for collaborative reflection and an opportunity to leverage insights from both traditional data and lived/living realities. The group sharing of lived/living experiences in a trusted and safe setting allows participants to process their experiences and support one another. In turn, this promotes both individual and group healing whilst simultaneously identifying meaningful opportunities for timely and equity‐oriented interventions for projects.

### 3.4 Step 3: Thinking About Change

Thinking about change builds on the above analysis. The community and Impact Partners identify specific changes that will (i) prevent the project from exacerbating existing inequities and (ii) make the project more inclusive and accessible to a wider group of people. This phase also involves multi‐level thinking, by encouraging all participants to explore change at the project, organizational and systemic level. It clarifies the roles of individuals, organizations and systems in implementing changes recommended by EMPaCT emphasizing collaborative accountability. The EMPaCT model focuses on identifying small, actionable changes that are feasible to implement, moving beyond ideological recommendations towards practical, achievable strategies that advance health equity incrementally. Participants also assess potential risks and benefits of the proposed changes to avoid unintended harm. Additionally, community members often facilitate projects by identifying resources, potential partnerships and networks that can support the recommended actions.

### 3.5 Step 4: Making and Communicating Recommendations

Step 4 transitions the co‐learning and collaborative problem‐solving of Steps 2 and 3 into actionable equity‐driven recommendations. This step takes place after Impact Partners leave the virtual meeting, allowing the community to engage in focused reflection and develop recommendations without the influence of potential power hierarchies. This independent space helps to amplify community insights by encouraging candid discussions about potential risks, gaps, and solutions. In addition, EMPaCT members can contribute through multimodal means—such as emails or asynchronous inputs—to respect their privacy and autonomy, enabling meaningful engagement without pressure to disclose personal experiences or participate in real‐time. Recommendations are typically categorized into two streams:

1. **Immediate actions**: These are feasible, small‐scale adjustments that align with the project's goals, timeline and resources. They focus on timely changes to prevent harm and promote equity.
2. **Long‐term opportunities**: These address systemic or structural issues that require sustained advocacy, resource mobilization and collaboration across stakeholders.

This approach balances short‐term actions with long‐term advocacy, promoting equity‐driven change across all levels.

### 3.6 Step 5: Closing the Loop With Reporting and Accountability

The concluding bookend of the EMPaCT CEn‐HEA focuses on documenting outcomes, tracking progress and creating feedback loops to uphold accountability. A confidential engagement report prepared for Impact Partners and validated by community members is the core of this step. This report summarizes key themes, recommendations and actionable next steps, serving as both a record of the engagement and a tool for tracking progress across multiple projects. It also supports collective capacity‐building, enabling community members to strengthen their knowledge, experience, and expertise through ongoing participation in equity‐driven initiatives. The engagement report is shared with Impact Partners within 4–6 weeks following the EMPaCT session. Impact Partners are asked to complete a post‐engagement feedback form to document changes they implemented based on the recommendations and to provide an explanation for changes they could not make. This reflective process helps clarify obstacles and opportunities, fostering a shared understanding of how recommendations are addressed and identifying areas for future focus. Post‐engagement debriefs are conducted with both community members and when needed, with Impact Partners to review challenges, assess successes and discuss areas for improvement. These sessions create an iterative feedback loop, where insights from one engagement inform and refine future practices. This process helps to continuously improve the EMPaCT model, fostering capacity and reinforcing its impact on health equity initiatives.

This step consolidates the work, promotes shared accountability, builds on the trust developed in earlier steps and supports incremental progress in advancing equitable practices within health systems and communities.

## 4 Tracking and Measuring Impact From EMPaCTs CEn‐HEAs

A limitation of traditional HEAs is the absence of frameworks to systematically track and monitor their long‐term impact. Often, HEAs focus narrowly on immediate outcomes, leaving systemic and structural changes inadequately assessed. To address this gap, EMPaCT has drawn on the Canadian Academy of Health Sciences model for evaluating research impact [35] to develop a comprehensive framework. This framework integrates capacity building, knowledge advancement and decision‐making impact to assess outcomes and promote accountability, ensuring that the transformative potential of CEn‐HEAs is fully realized.

### 4.1 Building Capacity

Capacity building focuses on the development of resources, networks and partnerships to sustain equity‐driven initiatives over time. EMPaCT's CEn‐HEAs prioritize building the infrastructure needed for ongoing engagement and long‐term progress. Metrics captured in this domain assess:

- Strengthening the skills and competencies of both community members and Impact Partners for meaningful participation in equity‐focused projects.
- Expanding the availability of resources that support sustained engagement in equity‐promoting activities.
- Creating sustainable partnerships that enhance the reach and influence of equity‐focused initiatives within and across communities.

### 4.2 Advancing Knowledge

Advancing knowledge reflects the ability of EMPaCT CEn‐HEAs to generate and disseminate actionable insights rooted in community engagement. EMPaCT prioritizes co‐created outputs tailored to the needs of the community, which contribute to public education and broader health equity discourses. Examples of knowledge advancement include:

- Developing co‐designed technical tools, frameworks and other resources to address equity challenges.
- Creating dissemination materials such as publications, reports, presentations and webinars that communicate findings effectively to diverse audiences.
- Broadening the understanding of health equity through publications, digital content and national/international dialogues informed by CEn‐HEA insights.

### 4.3 Informing Decisions

The ultimate aim of EMPaCT's CEn‐HEAs is to influence decision‐making processes at local, regional and national levels. This domain evaluates how community perspectives inform actions taken in response to CEn‐HEA recommendations. Metrics include:

- The extent to which diverse decision‐makers incorporate CEn‐HEA findings into policies, practices and research.
- Documented changes in programmes, policies or systemic approaches that align with priorities identified during CEn‐HEA sessions.
- Long‐term institutional shifts that address structural inequities and embed equity into organizational practices.

### 4.4 Feedback and Continuous Learning

In addition to these domains, EMPaCT integrates qualitative feedback from both community members and Impact Partners (data forthcoming) to capture the nuances of engagement. Insights from this feedback provide a deeper understanding of how the Five‐Step CEn‐HEA process is operationalized, offering reflections on challenges, successes and iterative learning that have shaped its development.

By systematically tracking capacity building, knowledge advancement and decision‐making impact, EMPaCT's evaluation framework demonstrates how the outcomes of individual CEn‐HEAs inform broader systemic changes. This approach places the lived/living experiences of community members at the centre of the evaluation processes, guiding the development of policies and programmes that are inclusive, equity‐driven and sustainable. In doing so, EMPaCT bridges the gap between immediate actions and long‐term transformations, fostering enduring progress toward health equity.

## 5 Conclusion

The co‐created EMPaCT CEn‐HEA model transforms traditional HEAs by centering lived/living experiences, embedding accountability and fostering equity throughout its five‐step process. By integrating robust methodologies such as beneficiary engagement and iterative learning cycles, EMPaCT bridges the gap between systemic inequities and actionable solutions. Aligned with the Canadian Academy of Health Sciences' framework, the model ensures measurable outcomes while advancing equity‐driven actions that reflect the needs of those most impacted. Through this participatory and transformative approach, EMPaCT redefines traditional HEAs into CEn‐HEAs that build trust, promote inclusivity, and drive sustainable change.

## Author Contributions

**Ambreen Sayani:** conceptualization, funding acquisition, writing ‐ original draft, writing ‐ review and editing, visualization, project administration, resources, supervision. **Linda Monteith:** conceptualization, writing ‐ review and editing. **Anam Shahil‐Feroz:** conceptualization, writing ‐review and editing, writing ‐ original draft. **Diya Srinivasan:** conceptualization, writing ‐ review and editing. **Isra Amsdr:** conceptualization, writing ‐ review and editing. **Fatah Awil:** conceptualization, writing ‐ review and editing. **Emily Cordeaux:** conceptualization, writing ‐ review and editing. **Victoria Garcia:** conceptualization, writing ‐ review and editing. **Ryan Hinds:** conceptualization, writing ‐ review and editing. **Tara Jeji:** conceptualization, writing ‐ review and editing. **Omar Khan:** conceptualization, writing ‐ review and editing. **Bee Lee:** conceptualization, writing ‐ review and editing. **Mursal Musawi:** conceptualization, writing ‐ review and editing. **Jill Robinson:** conceptualization, writing ‐ review and editing. **Staceyan Sterling:** conceptualization, writing ‐ review and editing. **Dean Wardak:** conceptualization, writing ‐ review and editing. **Kelly Wu:** conceptualization, writing ‐ review and editing. **Mohadessa Khawari:** conceptualization, writing ‐ review and editing. **Meghan Gilfoyle:** conceptualization, writing ‐ review and editing. **Alies Maybee:** conceptualization, writing ‐ original draft, writing ‐ review and editing, supervision.

## Ethics Statement

No ethics approval was needed for this commentary as no data was collected or analysed to inform the manuscript.

## Conflicts of Interest

Ambreen Sayani is a recipient of the Transition to Leadership Stream Career Development Award in Patient‐Oriented Research from the Canadian Institutes for Health Research and is a Health Equity Advisor to the Canadian Partnership Against Cancer (CPAC). All other authors declare no competing interests.

## Data Availability

Data sharing not applicable no new data generated.
